# An updated review of the genotoxicity of respirable crystalline silica

**DOI:** 10.1186/s12989-018-0259-z

**Published:** 2018-05-21

**Authors:** Paul J. A. Borm, Paul Fowler, David Kirkland

**Affiliations:** 1 Borm Nanoconsult Holding BV, Proost Willemstraat 1, 6231 CV Meerssen, The Netherlands; 2 FSToxconsulting Ltd., Raunds, UK; 3 Kirkland Consulting, Tadcaster, UK

**Keywords:** Crystalline silica, Quartz, Nanoparticles, Genotoxicity, Risk assessment

## Abstract

Human exposure to (certain forms of) crystalline silica (CS) potentially results in adverse effects on human health. Since 1997 IARC has classified CS as a Group 1 carcinogen [1], which was confirmed in a later review in 2012 [2]. The genotoxic potential and mode of genotoxic action of CS was not conclusive in either of the IARC reviews, although a proposal for mode of actions was made in an extensive review of the genotoxicity of CS by Borm, Tran and Donaldson in 2011 [3]. The present study identified 141 new papers from search strings related to genotoxicity of respirable CS (RCS) since 2011 and, of these, 17 relevant publications with genotoxicity data were included in this detailed review.

Studies on in vitro genotoxic endpoints primarily included micronucleus (MN) frequency and % fragmented DNA as measured in the comet assay, and were mostly negative, apart from two studies using primary or cultured macrophages. In vivo studies confirmed the role of persistent inflammation due to quartz surface toxicity leading to anti-oxidant responses in mice and rats, but DNA damage was only seen in rats. The role of surface characteristics was strengthened by in vitro and in vivo studies using aluminium or hydrophobic treatment to quench the silanol groups on the CS surface.

In conclusion, the different modes of action of RCS-induced genotoxicity have been evaluated in a series of independent, adequate studies since 2011. Earlier conclusions on the role of inflammation driven by quartz surface in genotoxic and carcinogenic effects after inhalation are confirmed and findings support a practical threshold. Whereas classic in vitro genotoxicity studies confirm an earlier no-observed effect level (NOEL) in cell cultures of 60-70 μg/cm^2^, transformation frequency in SHE cells suggests a lower threshold around 5 μg/cm^2^. Both levels are only achieved in vivo at doses (2–4 mg) beyond in vivo doses (> 200 μg) that cause persistent inflammation and tissue remodelling in the rat lung.

## Background

Prolonged chronic inhalation exposure to respirable crystalline silica (RCS) in the forms of quartz or cristobalite) can induce silicosis and, under certain circumstances, may also cause lung tumours. In 1997, the International Agency for Research on Cancer (IARC) classified crystalline silica (silica) as a Group I human carcinogen [1]. This classification was confirmed in a more recent review [2] but the pathogenesis of CS-induced lung cancer was not clearly identified, leaving many open questions about risk assessment of CS-containing particle exposure.

At the time of the IARC reviews, most genotoxicity assays with CS had been performed with quartz samples. Some studies gave positive (genotoxic) responses, but most were negative (non-genotoxic). Some quartz samples induced micronuclei (MN) in Syrian hamster embryo cells, Chinese hamster lung V79 cells and human embryonic lung Hel 299 cells, but not chromosomal aberrations (CA) in the same cell types. Two quartz samples induced morphological transformation in Syrian hamster embryo cells in vitro and five quartz samples induced transformation in BALB/c3T3 cells [3]. While quartz did not induce MN in mice in vivo, epithelial cells isolated from the lungs of rats exposed by the intratracheal route to quartz showed *hprt* gene mutations [4]. The effect was also seen in rats after administration of low-toxicity particles causing persistent pulmonary inflammation. In addition, inflammatory cells from the quartz-exposed rat lungs caused mutations in epithelial cells in vitro*,* although direct treatment of epithelial cells in vitro with quartz did not cause *hprt* mutations [4]. Tridymite had been tested in only one study, where it induced sister chromatid exchanges (SCE) in co-cultures of human lymphocytes and monocytes [5]. Only one human study measuring genotoxic endpoints in subjects exposed to dust containing CS, but with no indication of the level of exposure, was available for the IARC reviews; the study showed an increase in the levels of SCE and CA in peripheral blood lymphocytes [2].

The IARC evaluation of the carcinogenicity of RCS was based on sufficient evidence of tumour induction in animals (mainly in rats), and sufficient evidence of tumour induction in humans. In the 2012 review, IARC concluded that the rat lung tumour response to CS exposure was most likely a result of impairment of “alveolar-macrophage-mediated particle clearance thereby increasing persistence of silica in the lungs, which results in macrophage activation and the sustained release of chemokines and cytokines”. In rats, this “persistent inflammation is characterized by neutrophils that generate oxidants that induce genotoxicity, injury and proliferation of lung epithelial cells leading to the development of lung cancer” [2]. However, the possibility of CS surface-generated oxidants or a direct genotoxic effect could not be ruled out, and it was not known which of these mechanisms, if any, occur in humans.

In 2011 Borm et al. [6] wrote a comprehensive review to complement the IARC (1997) review [1] including more recent publications. They summarized and evaluated the most relevant publications on the in vitro and in vivo genotoxicity of CS. Borm et al. discussed the genotoxic mode of action (MoA) of CS in relation to its carcinogenic activity, and, consistent with the later IARC (2012) review [2], three possible MoAs were proposed:

- **Direct,** which would require RCS particles to enter the nucleus and interact directly with DNA, release of free radicals that damage DNA, or disruption of chromosome segregation during mitosis.
- **Indirect,** in which RCS depletes antioxidants, thus increasing steady-state endogenous oxidative damage, or increased oxidative damage arising from mitochondrial activity, inhibition of DNA repair etc.
- **Secondary**, in which RCS causes inflammation, and thus genotoxicity is mediated by e.g. phagocyte-derived oxidants.

A series of in vitro studies investigating induction of DNA stand breaks (comet assay) or MN had suggested that quartz induces DNA damage in the absence of cytotoxicity. However, there was no evidence that CS particles can enter the nucleus of target cells, and secondary genotoxicity due to physiological stress induced at high concentrations may explain these findings. Quartz particles have been observed inside A549 human lung epithelial cells [7] but not within the nucleus or mitochondria. In these studies, it appears that fixed cells were embedded in Epon™ (epoxy resin blend) and sectioned with an ultramicrotome before microscopic examination. However, whatever method (light microscopy, EM, confocal microscopy) has been used for such observations, concerns have been raised [8] that when sectioning embedded cells or tissue for microscopic analysis, it is possible that particles on the surface of a cell could be moved by the ultramicrotome and accidentally deposited in the cytoplasm – “drag effects”. The uptake of nanoparticles in the nucleus and mitochondria, such as reported for nanosize amorphous silica, is not considered relevant to this discussion, since these particles use either specific receptors or penetrate through very small (< 10 nm) nuclear pores [9], and RCS particles are not nanosized.

More important in this respect is that RCS particles have not been found within epithelial cells after in vivo exposure in either animals or humans despite numerous in vivo studies, with larger numbers of animals, using high doses of different particles. Lifetime exposure or follow-up of rats after inhalation [10] or instillation [11] did not observe such particles upon careful and multiple lung tissue investigations [12].

In the original review of Borm et al. [6] it was noted that at least a 5-fold higher dose of CS was required to reach a threshold for genotoxic effects than for pro-inflammatory effects in vivo. This ratio even increases to 60/120-fold if the deposition in the proximal alveolar region is considered [13]. These data strongly suggested that inflammation is the driving force for genotoxicity observed in vivo*,* and that primary genotoxicity of deposited CS would play a role only at very high, possibly implausible, exposures and deposited doses. Thus, Borm et al. posed the hypothesis that the overriding mechanism of CS genotoxicity is via inflammation-driven secondary genotoxicity [6].

It is interesting to note how the risk assessment is handled by different countries and committees. The Health Council of the Netherlands (DECOS) concluded that the carcinogenicity of quartz is mediated by a non-stochastic genotoxic mode of action, in which a long-term irritation leads to endogenous lipid peroxidation, which produces tissue damage causing release of reactive molecules [14]. Since the irritation precedes the genotoxicity, a threshold below which cancer risk can be considered nil is implicated, and the OEL for RCS was set later at 0,075 mg/m^3^. The UK Health and Safety Executive [15] confirmed that most standard tests on genotoxicity were negative, but that the process of inflammation may cause genotoxicity as a result of increased production of reactive oxygen species (ROS) leading to oxidative DNA damage. Since the extent of genotoxicity was directly related to the severity of inflammation, HSE concluded that it was most likely that CS was not a direct-acting genotoxic agent but could lead indirectly to genotoxicity as a secondary consequence of inflammation and as such would be associated with a threshold. The workplace exposure limit for RCS was set by HSE at 0.1 mg/m^3^. In a draft screening assessment of quartz and cristobalite, Environment Canada and Health Canada [16] concluded that the “vast majority of the positive genotoxicity assay results can be explained by the generation of reactive oxygen species, as demonstrated experimentally, where ROS scavenging prevents the genotoxicity.” Along with epidemiological and animal data on carcinogenicity and inflammatory responses they used a threshold approach to reach a limit of 25 μg/m^3^ for inhalation exposure to crystallina silica equivalent with the Canada Labour Code with an Occupational Exposure Limit of 25 μg/m^3^.

The purpose of the current paper is to review the evidence that has been published since 2011 on the three modes of action [2, 6] and to consider whether this requires modification of the current approach for risk assessment. Comments on silica nanoparticles are only included where they have relevance/meaning for the evaluation of RCS induced genotoxicity mechanisms.

## Methods

The terms used for the initial search of different databases, including PubMed, Toxline, CCRIS, NTP and ECHA, included the following:

- Quartz or crystalline silica + genotoxicity;
- Quartz or crystalline silica + mutation;
- Quartz or crystalline silica + chromosome;
- Quartz or crystalline silica + micronucleus;
- Quartz or crystalline silica + DNA repair;
- Quartz or crystalline silica + comet.

In addition, searches were performed on the same combination of these terms but also include inflammation, surface, toxicity and fibrosis to retrieve papers that indirectly discussed genotoxicity of RCS. The latter search strings produced a much larger number of hits consistent with our previous review [6]. For example, the number of hits in PubMed for fibrosis or toxicity or inflammation was 413, whereas the same search terms previously retrieved 376 references [6]. Table 1 shows the results of the searches on PubMed, Toxline and CCRIS, both unsorted (all results) and sorted (excluding what was non-relevant). We also searched NTP and ECHA (general search window), but these databases do not take Boolean search terms. Only one file was detected in the ECHA database, but no registration dossier was identified.

**Table 1:** The number of references retrieved from different databases for the search between 2011 and 2017 using specific combinations of search items relating to genotoxicity

Search item/Database Quartz or crystalline silica +	PubMed	ToxLine	CCRIS	Total
genotoxicity	18	2	0	20
mutation	32	58	0	90
chromosome	11	13	0	24
Micronucleus	10	1	1	12
DNA repair	2	8	0	10
Comet	12	0	0	12
Total (after exclusion of double hits)	**59**	**81**	**1**	**141**
Inflammation OR fibrosis OR toxicity	413	220	0	633
Surface	2929	392	0	3321

The search strings identified 141 papers published since 2011 (see Table 1). These were then filtered depending on whether the abstract contained any genotoxicity data from established hazard identification assays or mechanistic studies that could help explain the biological processes occurring after exposure to CS. Study quality (i.e. Klimisch score) was not considered relevant as the majority of studies were not performed according to GLP or regulatory guidance (for appropriate study types) but did contain potentially useful data for building a weight of evidence. In many papers CS has been used as a positive control in evaluation of the (geno) toxicity of other particles. These papers were considered relevant, and included in the review, but it is questionable whether the results of a study not specifically directed to the investigation of the substance should be considered with the same level of weight.

Of the papers selected for detailed review, 7 dealt with in vitro genotoxicity, 5 papers contained data on in vivo genotoxicity outcomes (one paper contained both in vitro and in vivo genotoxicity data). Five papers were classified as containing biologically relevant data, which would aid interpretation of the genotoxicity data, thus, of the 141 papers identified by the searches, 17 new publications were considered relevant and the review of the genotoxicity of RCS that follows was based on these.

### In vitro genotoxicity

A large portion of the in vitro studies only used RCS (mostly DQ12) as a reference material in their efforts to screen the genotoxicity of other particles. Nevertheless, these were included in the overview, but need to be considered with great care in the final evaluation. An overview of all in vitro studies is shown in Table 2, and a detailed presentation is given in the text below.

**Table 2:** Overview of in vitro genotoxicity outcomes obtained with crystalline silica of respirable size in in vitro studies

Sample characteristics	Test system	Outcomes	Reference
DQ12 quartz milled to 410 nm average diameter (100–800 nm). Used as positive control	Micronuclei (MN) in human PBL pooled from 2 donors (similar to OECD test guideline 487) after 24 h incubation in the absence of metabolic activation. Dose-response study.	No increase in MN in the concentration range testing at 32, 100, 320 and 1000 μg/ml. Particle number at lowest concentration was 5.32 × 10^9^	[17]
DQ12 quartz as positive control in evaluation bentonite genotoxicity. GSD: 2.0 μm, surface area: 5.7 m^2^/g Bentonite particles (BP; 6.8 and 6.5% CS). GSD 2.7 and 2.5 μm. Surface area: 60 and 108 m^2^/g	COMET and MN in Human B cell line (HM2.CIR), at concentrations 30–240 μg/ml COMET and MN in Human B cell line (HM2.CIR), at concentrations 30, 60, 180, 240 μg/ml	Slightly NS elevation of MN and COMET in cell line, after 24 h exposure to highest concentration (240 μg/ml). At 72 h of exposure all values are increased. Dose-dependent increase in MN and COMET above concentrations of 60 and 120 μg/ml, respectively; but only at 24 h. At 48 and 72 h all BP samples show increased MN and COMET. Bentonite particles show a stronger effect than DQ12 quartz.	[18]
Pure milled quartz from mineral source (respirable, surface area 4. 2 m^2^/g) and vitreous silica (respirable, 5.0 m^2^/g). Size range determined with SEM between 0.5 and 5 μm	Macrophage cell line (RAW 264.7) and epithelial cell line (A549) were incubated 4 or 24 h. Concentrations 5–80 μg/cm^2^. MN frequency and % tail DNA (comet) were assessed	Only quartz and not vitreous silica caused changes in % tail DNA, but exclusively in RAW cell line and not in A549 cells. The effect was visible both at 4 and 24 h. Particle uptake was assessed and noted to be similar in both cell types, although quantitative data are lacking	[19]
DQ12 (3 μm) as reference particles for fibre study to elucidate effect of mineral composition Quenching all particles with aluminium lactate	Primary rat alveolar macrophages, 2 h incubation with 200 μg/cm^2^ Primary rat alveolar macrophages, 2 h incubation with 200 μg/cm^2^	Tail intensity in comet assay was induced 20-fold over control in the presence of significant cytotoxicity induced by DQ12 Particle toxicity and tail moment induction was reduced to control levels by pre-incubation with aluminium lactate (100 uM)	[20] [20]
DQ12 quartz as positive control in study aiming to investigate metal nanoparticles.	ToxTracker reporter assay in mouse embryonic stem (mES) cells. GFP induction and cell viability were determined with flow cytometry. mES cells were exposed to quartz particles (6.25, 25, 50 and 100 μg/mL) for 24 h	DNA damage reporter (*Bscl2*) and the general stress reporter (*Btg2*) genes were not induced by treatment with any concentration of quartz. Only the anti-oxidant reporter gene was induced in a concentration dependent way. No genotoxicity was observed in mES cells using the comet assay.	[21]
Ground Min-U-Sil (CS) with mean size of 3.7 um and purity of 99.5% CS. Aged versus freshly ground CS	Human bronchial epithelial cells (BEAS-IIB) and lung cancer cells with altered (H460) or deficient (H1299) p53 expression	Freshly fractured or aged silica produced divergent cellular responses in certain downstream cellular events, including ROS production, apoptosis, cell cycle and chromosomal changes, and gene expression. Exposure to freshly fractured silica also resulted in a rise in aneuploidy in cancer cells with a significantly greater increase in p53-deficient cells	[22]
Quartz Q1 with mean size (D_50_) of 12.1 μm. 0.89 m^2^/gram and DQ12 (D_50_, 3 μm)) with or without different organosilane coatings (PTMO, SIVO 160) and Al-lactate as control inhibitor	Primary rat alveolar macrophages, 1.5–2 × 10^5^ cells per well, 2 h incubation with 75 μg/cm^2^	Both Q1 and DQ12 caused significant DNA damage in comet assay associated to cytotoxicity (LDH leakage). Both toxicity and DNA damage were blocked by pre-treatment of DQ12 with Al-lactate or organosilane compounds. Surface binding was found to be effective up to 168 h after treatment in artificial lysosomal fluid (pH 4.5).	[20]

Downs et al. [17] studied the induction of MN in vitro and in vivo by amorphous silica and gold nanoparticles, andDQ12 quartz was included as a reference control in both arms of the study. DQ12 was milled to reduce the average size to 410 nm (size range from 100 to 800 nm). Whole human donor blood was cultured in RPMI-1640 medium containing foetal bovine serum (FBS) and antibiotics, and the lymphocytes were stimulated to divide by addition of phytohaemagglutinin. After 44–48 h cells were centrifuged, re-suspended in fresh medium, and treated with a butanol suspension of DQ12 quartz at 4 different concentrations (32, 100, 320 and1000 μg/mL). Since cytochalasin B can interfere with the cellular uptake of nanoparticles, 2 treatment regimens were followed. For the first set of duplicate cultures cytochalasin B was added 4 h after the start of treatment, which then continued for a further 20 h. In the second set of cultures cytochalasin B was present for the full 24 h of DQ12 treatment. Cytotoxicity was determined using the cytokinesis block proliferation index (CBPI), and 1000 binucleate cells per culture (2000 per concentration) were scored for MN. Except for the lack of treatment in the presence of metabolic activation this design complies with OECD Test Guideline 487. Negative control MN frequencies were normal and were significantly increased by treatment with the positive control chemicals, mitomycin C and vinblastine. Following both treatment regimens, MN frequencies in DQ12 quartz-treated cultures were similar to those in vehicle controls and not significantly different. Interestingly, amorphous nanoparticles (15 and 55 nm) also did not induce MN at concentrations up to 1000 μg/mL.

Zhang et al. [18] compared the effects of native and active bentonite particles (containing 6.8 and 6.5% quartz respectively) as well as DQ12 quartz on induction of comets and MN in a cytochalasin B-blocked human B cell line (HM2.CIR). Active bentonite was obtained by treating native bentonite with H_2_SO_4_ (10–15%) causing a large specific surface area. Cells were exposed to the two samples of bentonite particles (BP) at concentrations ranging from 30 to 240 μg/mL for 3 different time periods, 24, 48 and 72 h. Concentrations were chosen based on absence of toxicity (trypan blue exclusion) from a separate experiment, but there were no concurrent toxicity measurements in the assays themselves and no indication of toxicity levels at tested concentrations. Additionally, 100 μL of supernatant from a formulation of 240 μg/mL was tested to assess genotoxicity of the water-soluble fraction and sampled at all 3 time points. For the MN test the frequencies of mononucleate, binucleate and multinucleate cells were not recorded, and so toxicity based on cell proliferation (e.g. CBPI) could not be established. MN in a total of 1000 binucleate cells per sample were scored, and whilst all slides were scored by the same individual it is unclear whether slides were coded to avoid bias. Treatment of cells with DQ12 quartz only, showed significant induction of both MN and %tail moment at high concentrations (240 μg/mL) and/or longer incubation periods (i.e. 72 h). In addition, the values for DQ12 MN frequencies were lower as compared to an equivalent particle mass of bentonite. MN frequencies at the highest concentration (240 μg/mL) after 72 h exposure were 6.00% MN for quartz and 8.25% MN for active bentonite compared to 1.67% in concurrent controls. Leaching of soluble components was indirectly excluded as a cause for bentonite genotoxicity. A possible explanation is that the difference in MN response is caused by a higher surface area in the BP samples or presence of small amounts of redox metals. However, no experiments were performed to investigate this option.

Guidi et al. [19] examined the genotoxicity of crystalline quartz (pure quartz flour obtained by grinding a very pure Madagascan crystal form in a ball mill), and vitreous (amorphous) silica generated by milling/grinding a very pure silica glass (Suprasil for optical applications), in an alveolar epithelial cell line (A549), and in a mouse macrophage cell line (RAW264.7). The comet assay and MN were used to estimate genotoxic potential, and trypan blue dye exclusion was used as a measure of toxicity at the start and end of treatments. Cultures were treated for 4 and 24 h with 5 different concentrations (plus appropriate controls) of quartz or vitreous silica (VS) ranging between 5 and 80 μg/cm^2^. In epithelial A549 cells both quartz and vitreous silica treatment showed little toxicity after 4 and 24-h exposure, with the highest concentration (80 μg/cm^2^). RAW264.7 cells were, however, more sensitive to toxicity induced by quartz, with negligible toxicity induced at the 4-h time point but a dose-related decrease in viability observed following 24 h treatment. The comet data showed small but statistically significant increases in % tail DNA in RAW264.7 cultures treated with quartz at all doses, a dose response relationship was not observed. All other combinations of cells and treatments did not increase the % tail DNA above concurrent controls. Quartz also induced reductions in proliferation of both RAW264.7 and A549 cells. On the other hand, VS induced an increase in proliferation in RAW264.7 cells. No increases in MN frequency by quartz or VS were noted inA549 or RAW264.7 cells. The different cellular responses were not explained by the ability to internalise particles since uptake was claimed to occur to the same degree in both cell types. Therefore, the different effect in A549 cells is suggested to be due to elevated antioxidant capacity or DNA repair efficiency [7] in A549 cells.

Ziemann et al. [20] investigated genotoxic effects from fibrous samples of alkaline earth silicate (AES) wools commonly used in furnace linings that under extreme heat generate cristobalite (a form of crystalline silica). DQ12 quartz and aluminium oxide were included as positive and negative particulate controls respectively. The tests were performed in primary rat alveolar macrophages, using a short treatment time of 2 h. The alkaline comet assay was used to evaluate genotoxic potential of all materials at a single concentration (200 μg/cm^2^). The comet assay was performed with and without several modifications, including modification with human 8-oxoguanine DNA glycosylase (hOGG1, which enhances breaks due to7,8-dihydro-8-oxoguanine, 8-oxoG) and aluminium lactate (AL; a purported “quencher” of biological effects from crystalline silica). DQ12 quartz induced a 20-fold increase in tail intensity over Al_2_O_3_ particle controls. Pre-treatment of quartz samples with AL completely inhibited the DNA-damaging potential of DQ12 as observed in the comet assay. The tail intensity of DQ12-treated cells was explained by the induction of oxidative damage by DQ12.

Karlsson et al. [21] used Toxtracker, a reporter based assay performed in mouse embryonic stem cells, using DQ12 as a reference particle in studying the effects of metal nanoparticles on the system. The reporter genes used were *Bscl2* (DNA damage via ATR pathway), *Srxn1* (*Nrf2* antioxidant response pathway for ROS) and *Btg2* (general stress via P53 pathway). Cells were treated with quartz particles for 24 h at concentrations of 6.2, 25, 50 and 100 μg/mL. Cell survival (reduction in cell number) was not affected at any of the tested concentrations. Data from the reporter assay showed that the DNA damage reporter (*Bscl2*) and the general stress reporter (*Btg2*) genes were not induced by treatment with any concentration of quartz. Only the antioxidant response (*Srxn1*) was induced in a concentration-dependent manner up to a maximum of threefold (relative to solvent controls) at the highest tested concentration of DQ12 (100 μg/mL). The advantage of this reporter based assay is the separation of genotoxic pathway responses from the antioxidant response indicative of elevated ROS. In vitro tests such as the micronucleus assay are often conducted in culture medium with low levels of ROS scavengers or antioxidants; as such they will result in positive responses due to ROS that do not occur in vivo due, in part to a far higher level of antioxidants. It is therefore useful to understand the contribution ROS makes to the in vitro response. In these mouse embryonic stem cells there appears to be no induction of DNA damage as measured by ATR pathway. Of course, it is possible that there may have been induction of gene expression at higher concentrations. On the other hand, the elevated ROS response indicates that treatments did produce effects, and reinforces the ROS-related effects seen from other studies.

Gwinn et al. [22] compared various molecular alterations induced by freshly fractured and aged CS (Min-U-Sil) in human immortalized/transformed bronchial epithelial cells (BEAS-IIB) and lung cancer cells with altered (H460) or deficient (H1299) p53 expression. This was of interest because there is strong evidence that fracturing silica results in the generation of increased levels of ROS on the cleavage planes, which can react with water to generate •OH radicals, and exposure to fractured silica produces enhanced lung injury and disease in exposed workers. As well as the molecular alterations, chromosomal aberrations were examined in cells that had been exposed to sieved fractured or aged silica at 500 μg/mL for 6 or 24 h. It appears that only a single experiment was performed, and only 50 cells per treatment were scored for aberrations. Significant increases in the percentage of cells with aberrations were seen at both 6 and 24 h in the H460 and H1299 cells including appearance of dicentric chromosomes. However, these cell lines do not have normal p53 function, although apoptosis was induced in these cells. By contrast, in the BEAS-IIB cells, which have normal p53 function, small increases in the percentage of aberrant cells were not significant. It should however be noted that with such small numbers of cells scored, these results are of questionable biological relevance. Aneuploidy, as measured by flow cytometry, was also induced in the H460 and H1299 cells exposed to both freshly fractured and aged silica, but was not induced in the BEAS-IIB cells. From these data it seems that normal healthy cells with functional p53 are not sensitive to ROS-induced structural or numerical chromosome damage.

Ziemann et al. [23] studied the cytotoxic and genotoxic effects of DQ12 quartz and new pure quartz (Q1) particles from the ceramic industry in primary rat alveolar macrophages. In the study they used several commercially available organosilane coatings to block the highly reactive silanol groups at the quartz surface. Both quartzes were coated with different amounts of hydrophobic DynasylanR PTMO (propyltrimethoxysilane) and hydrophilic DynasylanR SIVO 160 (oligomeric, amino-modified siloxane). Primary alveolar macrophages isolated from female Wistar rats by bronchoalveolar lavage were pre-cultured for 24 h, and then treated for 4 h in the dark. At the end of treatment, samples of culture supernatant were taken for measurement of LDH release (measure of cytotoxicity). For the alkaline comet assay, cells were placed on ice for 10 min to allow detachment from the culture vessel surface without using trypsin, which can cause membrane damage, these detached cells were analysed for DNA strand breaks. Both the test quartz (Q1) and DQ12 caused significant cytotoxicity and DNA damage at the test concentration of 77 μg/cm^2^. Both PTMO and SIVO 160 inhibited quartz-induced LDH release in a concentration dependent manner. Both PTMO and SIVO 160 clearly reduced the tail intensity of comets induced by Q1 and by DQ12, and thus reduced DNA damage. These data suggest that reduction in surface reactivity via organosilanes leads to a reduction in DNA damage. However, the chosen target cells are not relevant for fixing DNA damage into mutations (see below), and genotoxicity was not evaluated in the in vivo model reported in this paper which only investigated the effects of CS on lung organ weights and biochemical analysis of BAL fluid from treated male Wistar rats after 3 months intratracheal instillation with Quartz.

Interestingly most studies described above have used classical tests to measure DNA/chromosome damage (comet, MN) in primary or cultured macrophages. These cells play a role in the MoA to produce ROS and growth factors to create an environment for increased DNA damage, proliferation and fixation of damage. However, macrophages are not considered relevant as surrogate target cells to evaluate genotoxic damage to the lung [6]. In addition, MN and comet results do not correlate with particle induced carcinogenicity as demonstrated by Darne et al. [24]. They studied MN and comet induction in Syrian hamster embryo (SHE) cells, and in Chinese hamster lung (V79) cells, to evaluate the genotoxicity of quartz (Min-U-Sil, fully crystallised), commercial cristobalite (Chd, partly crystallised) and chrysotile (asbestos) as known carcinogens, and diatomaceous earth (DE, 100% amorphous) as control. The key data are summarised in Table 3. Reduction in cell number was used to estimate toxicity from both SHE and V79 cultures, and all silica species tested showed statistically significant reduction in cell numbers, with several materials inducing 50% toxicity and above in SHE cells at the highest tested concentration (amorphous, partly crystallized silica, and quartz induced 40–50% toxicity). However, in V79 cells, only amorphous silica induced more than 50% toxicity, whereas quartz and partly crystallised silica induced 35 and 25% toxicity respectively. Chrysotile was highly toxic to both cell types. The cell transformation data showed that amorphous silica (DE) did not induce morphological transformation (except for 3 colonies in one experiment at 15.24 μg/cm^2^), while a concentration-dependent increase in transformation frequency was induced by the two other silica samples. It should be noted that Min-U-Sil did not induce any cytotoxicity at concentrations up to 50 μg/cm^2^ but did induce morphological transformation in a dose-dependent manner. The partially crystalline commercial cristobalite was slightly cytotoxic and also induced transformation in a dose-dependent manner. Whereas cell transformation correlated well with carcinogenic hazard of the above materials, only the highest concentration of chrysotile showed a statistically significant increase in MN frequency in SHE cells (24 h). In V79 cells, statistically significant increases in MN frequency were caused by amorphous silica (therefore a “false positive” compared to cell transformation) and chrysotile. The comet assay (both at 3 and 24-h exposures) showed no statistically significant increases in % tail DNA except for the highest concentration of chrysotile in SHE cells treated for 24 h without FPG. Interestingly, trypan blue exclusion at both time points showed cellular toxicity of 80% and above in all treatments.

**Table 3:** Morphological transformation versus outcome in genotoxicity assays in Syrian hamster Embryo (SHE) cells treated with different forms of amorphous and crystalline silica

Test material	Dose (μg/cm^2^)	MN^1^ (% of cells) 24 h	Comet^1^ (% tail) + FPG, 24 h	Morphological transformation frequency (%)
Control	0	2.3 ± 0.5	13.0 ± 1.0	0.01
Diatomaceous earth (DE) (GSD: 1.35 μm)	3.81			0.02
	7.62			0.02
	11.4		11.0 ± 1.2	
	13.6	2.1 ± 0.8		
	15.24			0.11*
	22.8		18.0 ± 2.0	
	27.2	2.0 ± 0.6		
	30.48			0
	45.7		21.0 ± 2.5 *	
	54.4	1.8 ± 0.6		
Heated DE (see above), containing 47% Cristobalite (GSD: 4.85 μm)	3.81			0.16*
	7.62			0.19*
	11.4		6.5 ± 1.5	
	13.6	3.0 ± 1.0		
	15.24			0.23*
	22.75	2.0 ± 0.6	9.0 ± 1.0	
	30.48			0.4*
	45.7		10.0 ± 4.5	
	54.4	1.8 ± 0.3		
Quartz Min-U-Sil 5 (GSD: 1.33 μm)	3.81			0.24*
	7.62			0.17*
	11.4		9.8 ± 1.2	
	13.6	2.2 ± 1.3		
	15.24			0.71*
	22.8		11.0 ± 2.7	
	27.75	2.2 ± 1.3		
	30.48			0.77*
	45.7		8.0 ± 1.0	
	54.4	1.5 ± 0.3		

^1^Data were estimated from graphical representations in Darne et al [24] (Figures. two A (MN) and Figures. three B (Comet), reproduced with permission). *GSD* Geometric mean diameter

* = statistically significant (*p* < 0.05) compared to control

### In vivo genotoxicity (animal, human)

An overview of the papers included in this section is given in Table 4, including both animal and human data. As stated before in section A**,** Downs et al. [17] compared the induction of comets and MN in rats by amorphous silica and gold nanoparticles, and milled DQ12 quartz was used as a control for both parts of the study *(*in vitro*,* in vivo). Adult male Wistar rats were dosed intravenously with a suspension of quartz particles (400 nm, 100 mg/kg, 2 × 10 ^11^ particles, 3.12 10^9^ μm^2^) on 3 occasions, 48, 24 and 4 h before sacrifice. The dose of quartz (100 mg/kg) was the maximum tolerated dose (MTD), determined in a preliminary study. At termination, blood was removed from the abdominal *vena cava* for both MN and comet analysis. Whole body perfusion was performed on the animals, and the livers and lungs were separately perfused before standard processing for the alkaline comet assay [25, 26]. Comets (% tail DNA) were measured using an image analysis system from 150 nuclei for blood, liver and lung. For the MN assay in blood, fixed reticulocytes (RETs) were analysed using the Litron Microflow method [27, 28]. Cytotoxicity was determined from the percentage of RETs and the percentage of MN-RETs was determined in at least 10,000 RETs. For the comet assay, small (1.5–1.7-fold) increases in % tail DNA were seen in lungs, liver and blood of animals treated with DQ12 quartz, but none of these were statistically significant. No increases in the frequencies of MN-RETs were seen in quartz-treated animals. The weak genotoxic effects were only seen at the maximum tolerated dose in quartz-treated rats, and were accompanied by effects typically seen following inhalation of RCS such as neutrophilic infiltration, the occurrence of apoptotic cells, an increase in mitotic figures, and the induction of the inflammatory markers (TNF-αand IL-6) in plasma. Therefore, the authors concluded that the particle-induced tissue damage was probably mediated by an inflammatory response. However, the route of administration primarily served to match the purpose of the study, i.e. to study effects of nanoparticles after intravenous administration and therefore the data from this study is not considered appropriate for risk assessment purposes.

**Table 4:** Overview of genotoxicity outcomes obtained with crystalline silica of respirable size in animal or human studies

Sample characteristics	Test system	Outcomes	reference
DQ12 (410 nm) milled to reach this small size	Intravenous injection (tail vein), at a dose of 100 mg/kg to rats as reference to compare to nanosize silicas and gold nanoparticles. Acute effects up to 48 h.	MN and comets were measured in lung, liver and blood after 4 h, 24 h and 48 h. All effects seen were consistent with a secondary genotoxic mechanism: - No increased MN in circulating blood reticulocytes - No increased comets in blood cells - Increased plasma TNF-alpha - Increased apoptosis and liver inflammation after DQ12	[17]
Quartz Q1 with mean size (D_50_) of 12.1 μm. 0.89 m^2^/gram and DQ12 (D_50_, 3 μm)) with or without different organosilane coatings (PTMO, SIVO 160) and aluminium lactate as control inhibitor	90-day rat study. Particles (total dose 1 mg/animal), administered by intratracheal instillation of two 0.5 mg aliquots in 0.3 ml of PBS on consecutive days. Lavage and histology on day 28 and day 90.	Q1 and DQ12 both induced a persistent inflammatory response at 28 and 90 days. Both toxicity and inflammatory response were reduced to control levels by organosilane coatings of Q1. In vivo genotoxicty responses were not included in this paper.	[23]
DQ12 (respirable), 6 mg total cumulative dose	Instillation (intratracheal) with 3 × 2 mg each month	Poly (ADP-ribose), 8-OH-dG and OGGG 1 induction were assessed in lung tissue using immunohistochemistry (IHC). A good correlation was found between all genotoxicity markers and histopathological inflammation	[29]
DQ12, single dose of 100 mg/kg)	C57BL/6 wild-type (WT) and p47^phox−/−^mice; DQ12 administered by pharyngeal aspiration	In vivo oxidative DNA damage in lung tissue was not affected by quartz exposure and did not differ between p47phox− /− and WT mice. Neutrophils from the bone marrow of DQ12-treated WT mice, but not from p47^phox−/−^ mice, caused increased oxidative DNA damage when co-cultured with A549 epithelial cells.	[31]
Occupational exposure to respirable silica (< 70% RCS)	Male workers (*n* = 50) exposed to RCS dust in grinding, milling, bagging and sandblasting. Life time exposure by inhalation. Control group (*n* = 29) were office workers.	Both target cells (nasal epithelial cells, NEC) and non-target cells (PBL) were isolated and tested for percentage of MN. Increased MN were found in NEC (3-fold) and PBL (2-fold) of workers versus controls. A multiple regression analysis showed significant contribution of age, smoking and number of years exposure, particularly in workers’ target NEC but also in surrogate cells (PBL)	[32]

Rittinghausen et al. [29] used immunohistochemical methods to quantify various DNA damage markers, including 8-hydroxyguanosine (8-OH-dG) and 8-oxoguanine DNA glycosylase (OGG1), in the lungs of DQ12 exposed rats (intratracheal instillation once per month for 3 months to DQ12 quartz). The dose used induced nominal particle overload and persistent inflammation in the lungs. Nuclei containing 8-OH-dG and cytoplasmic areas containing OGG1 increased > 2-fold in the lungs of DQ12-treated rats. Levels of OGG1 in nuclei increased to a smaller but nonetheless significant amount. Such increases are indicative of oxidative damage to DNA. However, inflammation scores increased by > 6-fold. The levels of 8-OH-dG correlated significantly with the histopathologic inflammation scores, and the frequencies of OGG1-positive nuclei correlated, but not significantly, with the inflammation scores. As discussed earlier, oxidative damage and other genotoxic effects may well be driven by such inflammatory responses. However, oxidative stress can occur through direct generation of ROS as discussed earlier. Due to the severe particle overload in the lung in this study, secondary mechanisms, that may overwhelm and confuse potentially existing primary genotoxic events, prevented a clear distinction between the different primary and secondary genotoxic mechanisms in terms of the oxidative damage found in the rat lungs. It is interesting, however, that Møller et al. [30], in a review of the literature describing oxidative damage in animals exposed to particles, critically assessed the different ways in which oxidative damage to DNA could be measured as a marker of particle-induced genotoxicity in animal tissues. They considered assays of 8-oxo-7,8-dihydroguanine by antibodies (i.e. immunohistochemical methods), and/or unrealistically high background levels of 8-oxo-7,8-dihydroguanine, as non-optimal since these studies suggested experimental problems due to spurious oxidation of DNA. Such studies reported more induction of DNA damage after exposure to particles than did the publications based on more optimal methods such as the detection of FPG-sensitive sites, or where the number of oxidised guanine nucleobase lesions in control animals were below 10 lesions/10^6^ dG. Møller et al. [30] also carefully considered the routes of administration used, stating that studies using intra-tracheal instillation of particles into the airways of animals is a non-physiological procedure. Thus, the data of Rittinghausen et al. [29] would likely be considered non-optimal by Møller et al. [30]

Van Berlo et al. [31] investigated the contribution of phagocyte-derived ROS to inflammation, oxidative stress, and DNA damage responses 24 h after pharyngeal aspiration of DQ12 quartz (100 mg/kg) in the lungs of C57BL/6 wild-type and p47^phox−/−^mice, a mouse model featuring impaired phagocyte ROS generation due to knockout of the p47^phox^ NOX2 enzyme complex. Bone marrow-derived neutrophils were used for parallel in vitro investigations in co-culture with A549 human alveolar epithelial cells. DQ12 quartz induced a marked neutrophil influx in both wild-type and p47^phox−/−^mouse lungs. Significant increases in mRNA expression of the oxidative stress markers haemoxygenase-1 (HO-1) and γ-glutamylcysteine synthetase (γ –GCS) were observed only in quartz-treated wild-type animals. However, oxidative DNA damage, as measured by the FPG-modified comet assay, in lung tissue was not affected byDQ12 quartz exposure and did not differ between p47^phox−/−^ and wild type mice. Differences in mRNA expression of the DNA repair genes OGG1, APE-1, DNA Polβ, and XRCC1 were also absent**.**DQ12 quartz treatment of co-cultures containing wild-type neutrophils, but not p47^phox−/−^ neutrophils, caused increased oxidative DNA damage in A549 epithelial cell**s.** The authors concluded that neutrophil-derived ROS significantly contribute to pulmonary oxidative stress responses after acute DQ12 quartz exposure, but their role in induction of oxidative DNA damage could be shown only in vitro.

It is important to note that the ATR pathway examined by Karlsson et al. [21] detects single strand breaks is relevant for RCS because these lesions can be indirectly induced by ROS, but other pathways such as XRCC1 (BER) as investigated here [29’are probably not.

### Human data

One new paper described genotoxicity in humans exposed to RCS. Demircigil et al. [32] investigated the frequencies of MN in target nasal epithelial cells (NEC) and peripheral blood lymphocytes (PBL; effectively a surrogate tissue) of glass industry workers, sandblasters and stone grinders from 4 locations in Turkey. A total of 50 exposed males were selected, working mainly in grinding, mixing and bagging tasks. Mineralogical and elemental analysis indicated that the workers were exposed to dusts containing 70–100% CS. A control group of 29 males who were not exposed to the dusts were matched for age and smoking status. PBL were cultured using standard methods for 72 h, with cytochalasin B present for the final 28 h. NEC were obtained by scraping the inner nasal turbinate with a cytological brush, smearing the cells on to wet slides and allowing them to dry. Cells were fixed, stained with Feulgen and counterstained with Fast Green. For each individual 3000 nasal epithelial cells were scored for presence of MN.

The MN frequencies (from 1000 binucleate cells/individual) in the control peripheral blood samples (5.59 ± 2.86 MN/1000 binucleate cells) were in the normal range for young men in their thirties [33] According to Knasmueller et al. [34]. The control MN frequencies in nasal epithelial cells (from 3000 cells/individual) reported by Demircigil et al. [32] (2.84 ± 1.61 per 1000 cells) were the highest seen across 16 different published studies in this cell type. The lowest published control frequency was 0.14 MN/1000 cells. However, even considering the high background levels, MN frequencies in nasal epithelial cells were approximately 3-fold higher in workers than in controls, and the difference was statistically significant (*p* < 0.001). Moreover, a multiple linear regression (MLR) analysis of MN in NEC showed that the years of exposure was a highly significant descriptor (*P* < 0.001; B = 5.47) on top of smoking (*P* = 0.002) and age (*P* = 0.006). This clearly indicates a dose-response relationship suggesting direct genotoxicity; however such effects could be mediated by the high number of neutrophils (> 70%) present in nasal lining fluid.

In the peripheral blood lymphocytes, 2-fold increases in MN frequencies were found in workers compared to controls. Given that Fenech and Bonassi [33] report control MN frequencies in healthy young males up to 12/1000 binucleate cells, the levels seen in CS workers (also around 12/1000) may not be outside the normal range. A sophisticated MLR analysis of MN frequency on individuals versus years of exposure, age and smoking showed age and number of exposure years as significant descriptors (both *P* < 0.005), whereas smoking was of borderline (*P* = 0.087) significance.

If the increased MN frequencies in peripheral blood lymphocytes do represent a genotoxic effect, it could not be due to direct genotoxicity since RCS particles would not conceivably enter the blood or blood lymphocytes. Demircigil et al. [32] hypothesize that the MN could be caused by indirect or secondary effects such as reactive oxygen species or pulmonary inflammation as discussed earlier by Borm et al. [6]. An argument to support this mode of action is that levels of MN correlated well between PBL and NEC in the group of workers (*n* = 50).

### Other mode of action data

Fazzi et al. [35] investigated the different processes by which ROS are induced following phagocytosis of CS (α-quartz; average size 1.7 μm), by macrophages. Human monocytes, isolated from peripheral blood of normal human subjects, were differentiated into primary human macrophages. These, together with mouse bone marrow macrophages (from C57BL/6 J, BALB/c, and p47^phox**−**/**−**^ mice), and the macrophage cell lines RAW 264.7 and IC21, were used to study the contributions of NADPH oxidase (Phox) and mitochondrial ROS (mtROS) to CS-induced lung injury. 1 × 10^5^ macrophages were seeded into 96 well plates 24 h prior to exposure. Treatment was by media replacement with 20 μg/cm^2^ silica particles suspended in PBS for periods up to 6 h.

CS induces a rapid and sustained production of superoxide anion by RAW 264.7 (derived from BALB/c mice) macrophages, but the response is greater in IC21 macrophages (derived from C57BL/6 J mice). Consistent with this, in response to CS, p47^phox^ protein expression was decreased in IC21, but not in RAW 264.7 macrophages, even though baseline levels of Phox proteins were similar in the 2 cell lines. This reduced p47^phox^ expression in IC21 macrophages was linked to enhanced generation of mtROS, cardiolipin oxidation, and accumulation of cardiolipin hydrolysis products, culminating in cell death. The effect of CS on p47^phox^ protein expression in primary C57BL/6 J macrophages was similar to that seen in the IC21 macrophages, exhibiting a significantly decreased p47^phox^ protein expression (2–4 h). In contrast, no decrease in p47^phox^ protein expression was observed in BALB/c macrophages after CS exposure. The response to CS exposure in human macrophages was similar to that seen in C57BL/6 J macrophages, although decreased p47^phox^ protein expression was observed at later (4–8 h) times. The authors demonstrated that decreased p47^phox^expression was associated with increased mtROS (mitochondrial superoxide anion) production. This was also demonstrated in vivo in p47^phox−/−^ mice, which exhibit increased inflammation and fibrosis in the lung following CS exposure. CS induces interaction between TNFR1 and Phox in RAW 264.7 macrophages, and TNFR1 expression in mitochondria leads to decreased mtROS production and increased RAW264.7 macrophage survival. These results identify the TNFR1/Phox interaction as a key event in the pathogenesis of silicosis that prevents mtROS formation and reduces macrophage apoptosis. Thus, based on these data, and assuming human macrophages behave in a similar way to those in rodents, human macrophages would be expected to exhibit mtROS formation and apoptosis in response to CS exposure.

Pavan and Fubini [36] proposed an adverse outcome pathway for the induction of silicosis, autoimmune pathologies and lung cancer from inhaled CS. They noted that CS particles are cytotoxic, trigger the production of inflammatory substances, oxidants and growth factors from cells, and can damage membranes. However, when the same test was performed on a variety of silica samples the extent and even the nature of the cellular response varied. Coating of the quartz particles with aluminium, iron, and carbon sometimes led to enhanced effects, but sometimes led to reduced effects. Coating with polymers or other substances mainly led to reduced cellular responses. They discuss evidence [37] that implies the surface silanols are critical in the cellular reactivity of CS particles, and explain the above observations. Thus, they propose a pathway in which silanols available for H-bonding with external molecules lead to engulfment of coated particles by macrophages and a strong interaction of the uncoated particle with the phagolysosomal membrane leading to rupture, macrophage activation and inflammatory response. Whilst the data used in this study came from cells in vitro (fibroblasts and endothelial cells) and are therefore of questionable in vivo relevance, the data may still be useful in determining the molecular pathways initiated by CS exposure.

Several in vitro and *invivo* studies [20, 23] support this pathway since several agents known to bind to the silanol groups (aluminium lactate, organosilanes) were able to block cytotoxicity and inflammatory responses induced by DQ12 and workplace quartzes. Earlier work by Schins et al. [7], reviewed in Borm et al. [6], already showed the same for genotoxic outcomes after intratracheal administration of silicas.

Chan et al. [38] used RNA sequencing to evaluate the inflammatory and fibrotic effects of cultured A549 cells in vitro, exposed to CS up to a limit of 20% cytotoxicity. CS was 99% pure (Sigma, USA) and in the 0.5-10 μm size range. A549 cells were grown in DMEM supplemented with penicillin/streptomycin (1% solution) and 10% FBS. For cell treatments CS was suspended in DMEM with a lower level of FBS (1.25%), the original culture media was removed and replaced with DMEM containing CS. Treatment was for 0.5,2,8,16 and 24 h with a range of concentrations of CS (6.25–100 μg/cm^2^). Toxicity was estimated via cell counts (cell counting kit-8, Sigma USA) and a maximum level chosen for subsequent RNA sequencing that induced 20% cytotoxicity (12.5 μg/cm^2^ at 24 h exposure). For next generation sequencing (NGS), cells were treated with 12.5 μg/cm^2^ for 2,8,16 and 24 h. Cultures were washed and RNA extracted using RNeasy kits (Qiagen, USA), quality was assessed using a 2100 bioanalyser (Agilent, USA). The library was prepared according to the TruSeq RNA sample prep kit (illumina, USA) and quantitated on an Agilent bioanalyser (Agilent, USA). The libraries were then amplified on the cBot system (Illumina, USA) and sequenced on the HiSeq 2000 system (TruSeq SBS KIT-HS V3, Illumina, USA). The database for annotation visualisation and integrated discovery (DAVID, LHRI, USA) was used to generate a summary of enriched annotation terms. Gene network analysis was performed using the search tool for the retrieval of interacting genes (STRING database, ELIXIR data resources).

The authors found that CS induced 22 differentially expressed genes, 2 were up-regulated and 20 downregulated in all samples. The major transcriptional pathways with differential gene expression were well-documented silicosis pathways including oxidative stress and inflammation (data not shown). This further reinforces the MoA being primarily ROS induction as seen from the Toxtracker data of [21].

Gene expression was also investigated by Vuong et al. [39], alongside proteomic analysis, in cultured A549 cells in vitro after exposure to cristobalite and quartz (Min-U-Sil 5). Particles were prepared in buffer (0.19% NaCl, 25 μg/mL Tween-80) at a concentration of 10 mg/mL and diluted in culture media (DMEM) for treatment. 70% confluent flasks of A549 cells had culture media removed and replaced with dilutions of particulates in buffer (60, 140, 200 μg/cm^2^) prior to incubation for 24 h at 37 °C. At harvest, total protein and total RNA were extracted from treated and control cultures. For both silica samples, protein was analysed by 2D gel electrophoresis, identifying 49 protein spots, 30 of which had identities confirmed by MALDI-TOF analysis and 15 of these showed a dose related change in protein levels compared to untreated controls. Pathway analysis showed that all of the proteins with differential levels were associated with cell death, inflammation, homeostasis and proliferation pathways. Gene expression changes were evaluated using RT-PCR with a panel of 89 genes selected from previous literature on silica particle exposure as well as focusing on genes up and downstream of the protein pathways identified. They found 37 genes with differential expression post treatment with both particles compared to untreated controls. The majority of the genes with significant changes in expression were subsequently found to be involved with ROS, metabolism, homeostasis and inflammatory pathways (data not shown). Whilst the authors did not report any data from DNA reactivity or genotoxicity, the MoA of both cristobalite and Min-U-Sil appeared to be ROS and inflammation related, adding weight to the primary mechanism of toxicity after silica exposure being ROS related.

Pozzolini et al. [40] evaluated the role of surface radicals on cellular toxicity after exposure to unmodified quartz microcrystals (Min-U-Sil-5) and samples processed with ascorbic acid. Three different cell-lines, i.e. the mouse macrophage cell line RAW 264.7, isolated primary human dermal fibroblasts (neonatal foreskin) and an endothelial cell line (HUVEC) were exposed to modified and unmodified samples at a single, final concentration of 100 μg/mL. For cell viability assays the exposure was for 24 and 72 h at 37 °C, and viability was assessed via the MTT assay. ROS activity was measured in fibroblasts and endothelial cells, which were seeded the day prior to exposure and treated with a fluorescent dye (2,7-dichlor-dihydro-fluorescien diacetate, 10 μM) for 30 min prior to treatment with 100 μg/mL quartz (with and without ascorbic acid) which was present for 4 h before plates were read on a fluorescence plate reader (485 nm ex. 520 nm em.). Lipid peroxidation was measured using a spectrophotometric method (TBARS assay). Cells were prepared and treated as above, and exposure to both quartz samples was for 24 h at which point cells were washed, scraped off the surface of the plates and lysed. TBA solution was added in a 2:1 ratio (0.375% thiobarbituric acid, 15% trichoroacetic acid, 0.25 N HCl) for 45 min at 95 °C. After cooling 1 vol. of N-butanol was added and the organic phase read at 532 nm. At 24 and 72 h, fibroblasts showed no decrease in viability with both quartz varieties. However at 72 h there was a slight statistically significant increase in cell number in the presence of ascorbic acid modified quartz, suggesting stimulation of cell proliferation. Endothelial cells showed significant decreases in viability at 24 and 72 h in the presence of both quartz species. Macrophages did not show a reduction in viability at either time point with quartz but did show reduced cell viability from samples treated with ascorbic acid modified quartz (data not shown). Increases in measured ROS were seen after treatment of fibroblasts and endothelial cells with ascorbic acid modified quartz but not when using unmodified quartz. Fibroblasts showed a significantly higher production of ROS than endothelial cells (data not shown). Significant increases in lipid peroxidation were seen in both fibroblasts and endothelial cells treated with modified and unmodified quartz.

QPCR for a limited number of genes involved in inflammation (for endothelial cells) and anti-apoptotic and fibrotic pathways (for fibroblasts) were evaluated at 2 and 5 days post exposure. Fibroblasts showed increases in several genes with ascorbic acid modified quartz only. Endothelial cells also had upregulation in several genes however there were fewer differences between ascorbic acid modified and unmodified quartz.

The authors conclude that the intensity of cell responses is directly related to surface radicals on the quartz crystal, adding further weight to the evidence that the primary driver for toxicity in CS exposed cells is via ROS generation.

## Discussion

Reviews performed by panels in national [14–16] or international context [1, 2] have concluded that inhalation exposure to RCS can lead to lung cancer in humans based on sufficient evidence of tumour induction in animals and humans. In the updated 2012 IARC review, IARC concluded that the rat lung tumour response to CS exposure was most likely a result of impairment of “alveolar-macrophage-mediated particle clearance thereby increasing persistence of silica in the lungs, which results in macrophage activation, and the sustained release of chemokines and cytokines” [2].

At the same time, and consistent with the IARC (2012) review [2], Borm et al. [6] proposed 3 possible MoA; direct, indirect and secondary genotoxicity. However at the time there was insufficient data to make a clearer prediction on the most likely mechanism. Therefore in this paper we selected and reviewed a further 17 additional sources of data on CS genotoxicity, involving 7*in vitro* studies with RCS, 5 in vivo studies, 4 mechanistic investigations and a single human exposure study. These publications contain some novel mechanistic insights further supporting the view that the genotoxic MoA is explained by secondary effects from sustained inflammation, although some interesting new data on direct cell transformation are now available that forward further discussion. Before any quantitative comparisons are made, it is worthwhile to make a qualitative observation.

From the in vitro studies reviewed in this paper (Table 2) 5 out of 7 used (DQ12) quartz as a reference or positive control, and did not always focus on the genotoxicity of CS. When used as a reference material, less specific attention is paid to the concentrations and outcomes of the study. The use of RCS for i.v. injection as a positive control in [17] probably is the best example to what can happen if compounds are copy-pasted into studies simply to be used as positive control to other i.v. relevant agents, and not regarding the mechanisms that were extensively discussed [6].

The in vitro test data with CS particles show many inconsistencies in the number and quality of positive responses. This may be due to variation in concentrations of CS. Whereas some studies have used a wide concentration range [17], others have focussed on low-toxicity ranges [18, 21]. Most studies contained enough technical details to convert test concentrations to μg/cm^2^ revealing an average level of 75 μg/cm^2^ with 2 μg/cm^2^ as lowest [21] and 200 μg/cm^2^ [20] as highest concentrations.

As noted previously [6] the lowest level at which genotoxic effects are observed in vivo is around 40 μg/cm^2^. At this level most in vitro studies reviewed here showed negative outcomes, whereas only two studies [19, 23] noted DNA damage between 60 and 75 μg/cm^2^, in macrophages only. A dose of 40 μg/cm^2^ resulting from the inhalation of of 155 mg RCS in the rat, and can be derived from concentration multiplied by the rat alveolar surface (3880 cm^2^).Although there is variation with regard to surface with rodent age, far more important is deposition of particles in specific hot-spots. Therefore, we state that this dose (155 mg) is an overestimate and it would be better to use the surface of the proximal alveolar region (PAR). Assuming that the PAR is between 300 and 600 cm^2^ and all RCS is deposited in the PAR (worst case) an inhaled dose of 12–24 mg would be sufficient to reach this target concentration. The study by Darne et al. [24], reported morphological transformation in SHE cells around 5 μg/cm^2^ (see also Table 3). At this dose and up to 50 μg/cm^2^, MN and % DNA in the same cells were unaffected. When using this test and level as a critical endpoint, the calculated in vivo dose needed would be about 20 mg or 2–4 mg when including the PAR as a hot-spot region for deposit. This is still 10–15 times the inhaled doses (200 μg/rat) that were shown to cause persistent inflammation in sub-chronic exposure of rats to RCS [6]. For comparison the calculated concentration to initiate an inflammatory response, using the same estimates and assumptions, is between 0.3 and 0.7 μg/cm^2^. The safety margin between the calculated NOEL for lung inflammation (0.3–0.7 μg/cm^2^) and cell transformation (> 5 μg/cm^2^) is around a factor of 10.

It is well documented that different cell types have varying susceptibility to genotoxic insult, influenced largely by age (passage number), genome stability, p53 status, rodent or human origin etc. [41–43]. The in vitro data reviewed here (Table 2) show positive effects predominantly in macrophages. Macrophages, that are a crucial mediator of the inflammatory MoA, are also considered as a target cell of direct genotoxic outcome. Although their role is crucial (confirmed by Fazzi et al. [35]), utmost care should be taken when interpreting these data regarding mutagenicity and carcinogenic hazard of RCS, since genotoxic damage in macrophages will not lead to mutations in the lung epithelium. As it happens, many of the in vitro studies show no genotoxic effects of CS at all, and in those where increases in DNA/chromosome damage were seen, the effects were predominantly weak. Freshly fractured and aged silica induced structural chromosomal aberrations and aneuploidy in p53-defective human lung cancer cells but not in p53-efficient BEAS-IIB cells [22]. Quartz (but not amorphous silica) induced comets in mouse macrophages (RAW264.7 cells), where significant toxicity was induced, but not in A549 cells where toxicity was not induced [19], but there were no increases in MN with either cell type. Amorphous silica induced MN in V79 cells, but not in SHE cells, whereas quartz and cristobalite did not induce MN even at concentrations producing at least 40% toxicity. Amorphous silica also induced comets in V79 cells at the highest concentration tested (which induced 60% toxicity) and only after 24 h exposure [24]. Morphological transformation of SHE cells was reported by Darne et al. [24] for partially crystallised cristobalite and fully crystallised quartz (Table 3), in the latter case at cytotoxic doses, but such responses could be due to non-genotoxic effects that can be detected in the SHE cell transformation system (see Corvi et al. [44]). In this respect, the study by Darne et al. [24] is considered very important regarding the predictive value of classic genotoxicity tests (MN, comet) with regard to in vivo carcinogenicity.

Apart from cell types used, it is important to note that the combination of dispersion of particles and cell culture medium determines the number and size of particle (aggregates) to which the cells are exposed. As shown by Hadrup et al. [45] these effects were significant in genotoxicity outcomes for hydrophobic particles (carbon black, carbon nanotubes) but not for TiO_2_, which we consider to be more like the quartz with regard to hydrophilic surface. On the other hand, other studies [7, 46, 47] demonstrated that the quartz surface can be shielded or modified by agents in the mineral matrix, method of isolation from the matrix (crushing, grinding) or even presence of cations in the medium such as aluminium and PVNO [7]. Therefore, any quantitative comparison between in vitro genotoxicty outcomes of RCS is limited not only by the source, but also the pre-treatment and addition of the sample to the cell culture medium. This is a major difference to the in vivo studies where only source and dose are important.

The recent in vivo data (overview in Table 4) also support an indirect MoA with regard to genotoxic effects noted in animals and humans. DQ12 induced small increases in comets in blood, livers and lungs of rats dosed intravenously at the maximum tolerable dose. Also using this unusual route of administration (at least for assessment of particle toxicity), the responses were concluded to be due to marked inflammatory responses and there was no induction of MN in blood reticulocytes [17]. DQ12 quartz induced (oxidative) lung damage in several sub-chronic and chronic studies in rats using intratracheal instillation. In all instances a persistent pulmonary inflammation was observed both by lavage and by immunohistochemistry [20, 29]. On the other hand, DQ12 did not induce oxidative damage in the lungs of wild-type and NOX2-deficient mice, even though marked neutrophil influx was observed, and increased expression of oxidative stress markers was seen in wild-type mice [31]. This is in line with earlier reports [48, 49] showing that tumour induction by particles is much less pronounced or absent in mice, hamsters, guinea pigs and rabbits. Part of this may be related to a much more pronounced anti-oxidant response in these species as noted by van Berlo et al. [31].

As discussed previously [6, 7, 46] the role of the quartz surface is demonstrated to be crucial in the response to CS. Both in vitro [7, 22, 23] and in vivo studies [23] confirmed the crucial role of the silica surface. Freshly fractured or aged silica produced divergent cellular responses in certain downstream cellular events, including ROS production, apoptosis, cell cycle and chromosomal changes, and gene expression [22]. Pavan and Fubini [36] also propose an adverse outcome pathway for quartz toxicity based on the surface as a driver for toxicity and inflammation. The primary source of the genetic damage in their model is again from persistent inflammation at the site of exposure rather than the relatively small levels of ROS generated from the quartz itself.

There are also clear differences between toxicological effects depending on the method of isolation of RCS from the mineral matrix itself. Miles et al. [45] showed that naturally occurring quartz with occluded crystal surface resulted in significantly less inflammation then crushed reference quartz (DQ12) from 28 and 90 days after intratracheal instillations in rats. This could partly explain the differences observed between published studies and highlights the importance of careful physiochemical characterisation of CS samples used in experimental studies.

Surface treatment of different quartz species with aluminum lactate or hydrophobic coatings reduced both in vitro and in vivo toxicity, in vitro genotoxicity and in vivo inflammation [23]. This confirms earlier findings by Schins et al. [7] and Scherbarth et al. [12] using similar coatings and in vitro and In vivo models. Interestingly, studies by the same group [50] also suggested a differential effect of surface on cellular uptake. Cellular uptake is important to evaluate the observed DNA damage regarding the MoA of RCS. Silica particles need to be internalised into the cell and reach the nucleus where they can either interact with DNA directly, or even interfere with the mechanics of mitotic segregation leading to chromosome breaks or chromosome loss. Whilst there is evidence of induction of DNA strand breaks and chromosome damage, particularly in vitro as seen from the various comet and MN studies, there are several reasons why these responses are likely to be due to indirect or secondary effects. Several studies in this review have specifically addressed the uptake issue and could not demonstrate uptake of RCS in the nucleus, although uptake in the cytoplasm was noted (e.g. [19]). In vivo data [29] could not demonstrate uptake of RCS in epithelial cells. It was also shown that dose-dependent oxidative DNA damage by quartz (20–100 μg/cm^2^) may occur in epithelial cells without entering the nucleus of Type II cells as evidenced by transmission electron microscopy. Moreover, this damage could be blocked by mitochondrial electron transport inhibitors [51]. Many of the studies assessing the genotoxicity of mineral fibres used extended exposure times (up to 72 h) before chromosome damage was seen, and therefore it may take longer than 24 h for the cells to internalise larger particulates, depending on cell type [52]. Doak et al. [53] also discussed the effects cytochalasin B can potentially have on cellular uptake in the MN test. Most of the in vitro studies in this review included wash off of the particle treatment followed by a recovery phase in the presence of cytochalasin B. At the time of cytochalasin B addition, the cells would have sufficiently internalised CS particles. Thus, despite most MN studies not being conducted to current OECD guidelines there is adequate evidence that the test conditions would have resulted in intracellular exposures. Finally, a biomarker study in 50 workers versus 29 controls [32] supports the fact that inhalation of RCS can cause genotoxic effects both in target nasal epithelial cells, and surrogate blood lymphocyte cells. The statistical analysis of MN frequency clearly relationship to occupational exposure, age and smoking in this cohort. However, also here the increased levels are considered to be due to indirect effects such as (nasal) inflammation.

As already indicated above we consider the effects of silica nanoparticles as not relevant for the genotoxic properties of RCS. The main reason is that surface characteristics (e.g. surface area) of these NP are completely different. While amorphous silica’s are mostly soluble and uptake mechanisms and intracellular transport are highly different [9] It is recommended to conduct a separate review and risk assessment of nanoparticle induced genotoxicity and to evaluate mechanisms and particle characteristics that are important in NP induced genotoxicity.

## Conclusions

Overall, the data in this review confirm earlier findings that RCS can induce weak genotoxic effects in vitro, mostly in macrophages. These in vitro effects are most likely explained by intracellular ROS generation induced during particle uptake in the cell cytoplasm, which has only been observed in vitro. The in vivo studies discussed here, along with previous work, confirm that the organ damage and genotoxic effects are caused by the inflammatory status. Considering the accumulating evidence for a secondary genotoxic mode of action in vivo, and an indirect action in vitro, it is recommended to focus on international harmonisation of occupational exposure limits, accepting a safe threshold limit. For this purpose additional research should focus on the difference between rodent and human mechanisms in RCS induced ROS generation and defence. We also recommend to further explore the use of RCS surface activity as a surrogate metric to rank hazards of the multiplicity of RCS containing samples and products.

## Abbreviations

8-oxodG: 8-oxo-7,8-dihydro-2′-deoxyguanosine
8-oxoG: 7,8-dihydro-8-oxoguanine
BSA: Bovine serum albumin
CA: Chromosomal aberrations
CBPI: Cytokinesis block proliferation index
CS: Crystalline silica
CTA: Cell transformation assay
dG: Deoxyguanosine
DPBS: Dulbecco’s phosphate buffered saline
FBS: Foetal bovine serum
FPG: Formamidopyridine glycosylase
i.v.: Intravenous
LDH: Lactate dehydrogenase
MI: Mitotic index
MN: Micronucleus or micronuclei
MoA: Mode of action
MTD: Maximum tolerated dose
OGG1: 8-oxoguanine DNA glycosylase
PHA: Phytohaemagglutinin
RI: Replication index
ROS: Reactive oxygen species
SCE: Sister chromatid exchange
